# A human working memory advantage for social network information

**DOI:** 10.1098/rspb.2024.1930

**Published:** 2024-12-11

**Authors:** Jack L. Andrews, Karina Grunewald, Susanne Schweizer

**Affiliations:** ^1^ School of Psychology, University of New South Wales, Sydney, New South Wales, Australia; ^2^ Department of Experimental Psychology, University of Oxford, Oxford, UK; ^3^ Department of Psychology, University of Cambridge, Cambridge, UK

**Keywords:** working memory, social cognition, social networks, depression

## Abstract

As a social species, humans live in complexly bounded social groups. In order to navigate these networks, humans rely on a set of social–cognitive processes, including social working memory. Here, we designed a novel network memory task to study working memory for social versus non-social network information across 241 participants (18–65 years) in a tightly controlled, preregistered study. We show that humans demonstrate a working memory advantage for social, relative to non-social, network information. We also observed a self-relevant positivity bias, but an ‘other’ negativity bias. These findings are interpreted in the context of an evolutionary need to belong to one’s social group, to identify risks to one’s social safety and to appropriately track one’s social status within a complex network of social relationships.

## 1. Introduction

Humans are innately social [1]. We live in large, complex and dynamic social groups. These groups, or networks, are comprised of relationships between people who vary in their nature, quality and duration. Each of these relationships can have lasting effects on our health and well-being [2–5]. To successfully navigate these social networks, a sophisticated set of social cognitive processes is required [6].

One key process supporting the success of our social interactions is working memory. Acknowledging the lack of a uniformly agreed upon definition of working memory [7], here, we refer to it as a cognitive system that holds limited amounts of information in mind in the service of higher cognitive functions and action. Social working memory, the ability to hold multiple pieces of social information in mind, in particular, may be critical for navigating our social environments. Indeed, research has shown that the size of individuals’ social network correlated positively with their social working memory capacity [8]. These findings support the view that human social working memory emerged in response to expanding social networks and the cognitive demands these present [8,9].

Social network information is inherently relational in nature. In a social network, individuals can be represented as nodes, and their relationships with others in a network as ties, or edges [10]. Some non-social information can also be relationally represented (e.g. transport routes). Interestingly, humans appear to learn about relationally presented information in social networks more easily compared with information presented in non-social networks [11]. Brain imaging studies show that social and non-social memory processing more generally relies on distinct brain systems [12–14]. For example, researchers have found that social working memory may rely on two functionally distinct neurocognitive networks—one typically associated with cognitive and non-social working memory performance (canonical working memory network) and one typically associated with performance when thinking about people’s traits, beliefs and intentions (mentalizing network [15]). In line with these findings, social working memory has been proposed to be distinct from its non-social counterpart [15]. Working memory for social networks, then, may be distinct from working memory for non-social networks, though this remains empirically untested.

A core function of social working memory may be to continuously maintain and update information about whom to approach and especially who to avoid in novel social contexts. In line with this argument negative, relative to positive, social information appears preferentially processed and transmitted in social networks [16]. More generally, a negativity bias has been shown in other domains of cognition, including learning and decision-making [17,18]. The evolutionary account of a negativity bias in cognition proposes the bias evolved to confer an adaptive advantage by sensitizing humans to potential risks in their environments [19]. The capacity to update information about who to avoid, and indeed who others are avoiding, is adaptive, as involvement in or exposure to interpersonal conflict is associated with a range of adverse health outcomes [20–22]. Negative social relational information then may be maintained in working memory more readily compared with positive information.

This negativity bias for negative social information is particularly evident in individuals with depression, who show both increased attention toward negative social information [23] and a tendency to interpret ambiguous social information negatively [24]. The social risk hypothesis [25] of depression proposes that depressed states facilitate hypersensitivity to ambiguous and negative social information to reduce the risk of social rejection, which itself is a threat to mental health and humans’ basic need of social belonging [26]. Therefore, the hypothesis argues depression evolved in order to promote belonging by sensitizing individuals to the (lack of) value that they offer to their group and by enhancing their sensitivity to signs of social rejection that may put them at risk of social exclusion and its associated risks to survival [25]. This sensitization to potential social threats may, therefore, be associated with relatively better working memory for negative compared with positive social information in individuals with depression.

**Figure 1.**
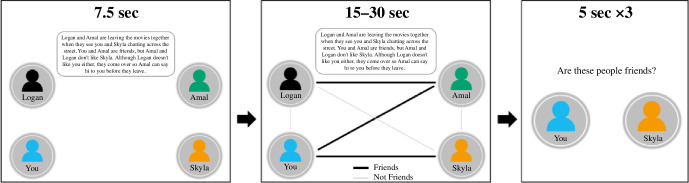
Network memory task. The figure illustrates a trial from the social-self condition. In each trial participants are first shown a vignette describing the relationships within the network for 7.5 s, a network diagram of these relationships then appears below the vignette. The diagram is shown for 15–30 s (i.e. participants have the option to proceed with the trial after seeing the diagram for a minimum of 15 s). Lastly, participants are shown three individual connections from the network and are asked if the people/flights shown are friends/operating (social/non-social conditions). Time spent viewing the diagram did not significantly differ (*F* = 2.11, d.f. = 479.15, *p* = 0.123 across conditions; non-social: *M* = 18.46, s.d. = 2.00; social-other: *M* = 18.68, s.d. = 2.02; social-self: *M* = 18.50, s.d. = 2.04).

Here, we designed a novel network memory task (NMT) to study working memory for social versus non-social network information across 241 participants (18–65 years) in a tightly controlled, preregistered study (https://osf.io/4fcxn). The task included three conditions: a self-relevant social condition (social-self), where participants memorized social networks that included themselves; a self-irrelevant social condition with networks including only other people (social-other); and a non-social condition that included flight networks between cities (figure 1). In each condition, participants were shown vignettes with corresponding network schematics describing the relationships between three (low cognitive load) or four (high cognitive load) different individuals or cities. Participants were then asked to identify whether connections from these networks were positive (individuals were friends/flights were running between cities) or negative (individuals were not friends/flights were not running between cities). The design allows us to dissociate social and non-social working memory. Given that personal social networks are likely to be more salient, and thus preferentially processed [27], we also contrasted domain-general social effects (i.e. observed across both social conditions) from self-relevant social effects (i.e. differing between social networks that include the self and social networks including only others). The task also allowed us to test for a negativity bias in social working memory by examining whether working memory for negative relations in networks is better remembered than positive relations. Finally, the association between depressive symptoms and working memory for positive and negative social versus non-social relations was examined across the networks.

A preregistered analysis plan tested the following hypotheses:

1. Working memory for relations from social networks will be remembered more quickly and accurately compared with relations from non-social networks (H1a). Negative relations will be remembered more quickly and accurately than positive associations (i.e. negativity bias (H1b)); and this negativity bias will be stronger in the social compared with the non-social conditions (H1c).
2. Individuals with high levels of depressive symptoms will show slower and less accurate working memory compared with individuals with low levels of depressive symptoms (H2a). Relative to the low depression group, the high depression group will more quickly and accurately classify negative versus positive associations (H2b), especially in the social compared with the non-social condition (H2c); and this effect will be strongest in the social-self condition (H2d).

## 2. Results

### (a) Sample and model overview

Participants (*n* = 241) were recruited through Prolific academic and aged between 18 and 65 years. Participant characteristics are reported in table 1 (for means and standard deviations of variables of interest, see electronic supplementary material, table S1).

**Table 1.** Sample characteristics ('training' refers to professional/vocational training; 'wealth' denotes self-perceived wealth).

	mean (s.d.)
**age (years**)	34.37 (10.92)
	***n (%)***
**gender**	
female	143 (59.34)
male	96 (39.83)
non-binary	2 (0.83)
different term	0 (0)
prefer not to say	0 (0)
**ethnicity**	
Aboriginal/Torres Strait Islander	3 (1.24)
African	15 (6.22)
Asian	40 (16.60)
caucasian	156 (64.73)
Hispanic	7 (2.90)
mixed	11 (4.56)
prefer not to say	3 (1.24)
other	6 (2.49)
**highest education**	
primary school	0 (0)
high school	57 (23.65)
training	33 (13.69)
university	151 (62.66)
**wealth**	
not at all	23 (9.54)
not very	85 (35.27)
fairly	108 (44.81)
rather	24 (9.96)
very	1 (0.41)

Validating the working memory paradigm, there was a significant effect of load (electronic supplementary material, table S2). In line with other working memory paradigms [7], participants recalled network associations faster (electronic supplementary material, figure S2a; *F* = 1129.61, d.f. *=* 1202.00, *p <* 0.001) and more accurately (electronic supplementary material, figure S2b; *F* = 439.29, d.f. *=* 1204.00, *p <* 0.001) under low cognitive load (networks made up of three people/cities) compared with high cognitive load (networks made up of four people/cities).

Working memory for networks also showed small, significant positive associations (all *r* = 0.17–0.21, all *p* < 0.05) with individuals’ performance in the affective condition of an affective backward digit span task (electronic supplementary material, table S3), a measure of working memory in emotionally salient contexts.

### (b) Working memory for social versus non-social networks

As predicted, working memory was better for social compared with non-social networks. That is, participants were both more accurate and faster at recalling relations from social compared with non-social networks (table 2*a*), as indicated by a significant main effect of condition (social versus non-social) in a linear mixed model including participant ID as a random effect (this was included in all mixed models and is not explicitly noted hereafter). All models also included age as a pre-registered covariate, due to age-related differences in working memory performance [28] and social information processing [29].

**Table 2.** Working memory for relations in social and non-social networks. Bold text denotes statistically significant results.

	reaction time				accuracy			
*predictors*	d.f.	*F*	*p*	*R* ^2^ *m/R* ^2^ *c*	d.f.	*F*	*p*	*R^2^m/R^2^c*
***(a)*** *social versus non-social (condition)*								
				0.12/0.79				0.03/0.47
condition	**480.17**	**47.69**	**<0.001**		**481.00**	**24.63**	**<0.001**	
age	**239.06**	**35.04**	**<0.001**		239.00	3.05	0.082	
***(b)*** *social-self, social-other versus non-social (condition)*								
				0.12/0.79				0.04/0.49
condition	**479.09**	**27.21**	**<0.001**		**480.00**	**23.06**	**<0.001**	
age	**239.06**	**35.04**	**<0.001**		239.00	3.05	0.082	
***(c)*** *positive versus negative relations (valence)*								
				0.10/0.72				0.01/0.43
valence	1202.00	0.52	0.471		1204.00	0.22	.640	
age	**239.05**	**35.39**	**<0.001**		239.00	3.05	0.082	
***(d)*** *social versus non-social (condition) and positive and negative relations (valence)*								
				0.11/0.73				0.02/0.45
condition	**1200.16**	**17.98**	**<0.001**		**1202.00**	**15.52**	**<0.001**	
valence	**1200.00**	**4.63**	**0.032**		1202.00	0.10	0.746	
age	**239.05**	**35.30**	**<0.001**		239.00	3.05	0.082	
condition × valence	**1200.00**	**4.45**	**0.035**		1202.00	0.54	0.464	
***(e)** social-self, social-other versus non-social (condition) and positive and negative relations (valence)*								
				0.12/0.75				0.04/0.46
condition	**1198.07**	**15.90**	**<0.001**		**1200.00**	**11.43**	**<0.001**	
valence	**1198.00**	**4.91**	**0.027**		1200.00	0.11	0.743	
age	**239.04**	**35.30**	**<0.001**		239.00	3.05	0.082	
condition × valence	**1198.00**	**34.82**	**<0.001**		1200.00	2.28	0.102	

**Figure 2.**
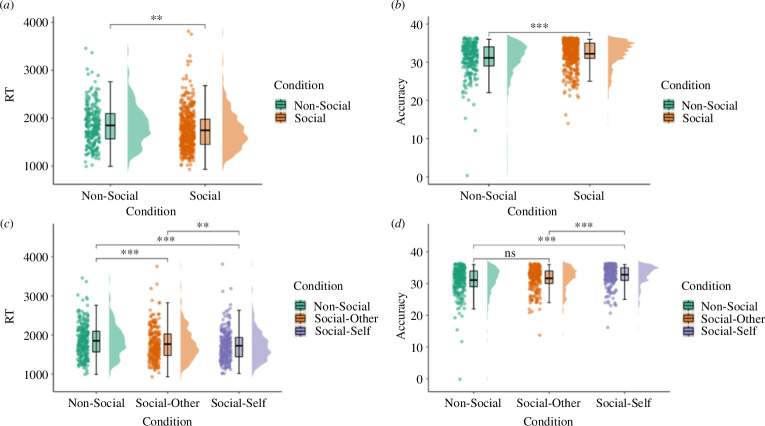
Network task performance depending on condition. (*a*) Reaction time performance across task conditions (non-social versus social). Panel (*b*) Total number of network associations recalled correctly across conditions (non-social versus social). (*c*) Reaction time performance across task conditions (non-social versus social-other versus social-self). Panel (*d*) Total number of network associations recalled correctly across conditions (non-social versus social-other versus social-self). RT = average reaction time on correct trials; Accuracy = number of trials correct out of 36 total/condition; Non-Social = trials measuring working memory for non-social relations (i.e. flight paths); Social-Other = trials measuring working memory for social relations in networks including only other individuals; Social-Self = trials measuring working memory for relations in social networks including the self and others; Social = trials measuring working memory for relations collapsed across social-other and social-self trials. *p < 0.05, **p < 0.01, ***p < 0.001.

Comparing working memory for non-social networks to social networks that included the self versus those that included only others revealed a self-reference effect (table 2*b*). That is, pairwise comparisons (electronic supplementary material, table S4) showed that participants recalled network associations faster (figure 2*c*) and more accurately (figure 2*d*) when they themselves were part of the network compared with social networks made up of others (accuracy: *β* = −0.30, 95% CI: −0.45 to −0.14, *t* = −4.53, *p* < 0.001; reaction time: *β* = 0.10, 95% CI: 0.00–0.20, *t* = 2.49, *p* = 0.038) and non-social networks (accuracy: *β* = −0.43, 95% CI: −0.59 to −0.28, *t* = −6.65, *p* < 0.001; reaction time: *β* = 0.30, 95% CI: 0.20–0.40, *t* = 7.26, *p* < 0.001). Participants were also faster at recalling network association when networks included others compared with non-social networks (*β* = 0.20, 95% CI: 0.11–0.27, *t* = 4.77, *p* < 0.001), although no difference was found for accuracy (*p* = 0.100).

Together, these results indicate working memory is faster and more accurate for relational information presented in social compared with non-social networks, particularly when this information pertains to the self.

### (c) Working memory for positive and negative social and non-social relations

**Figure 3.**
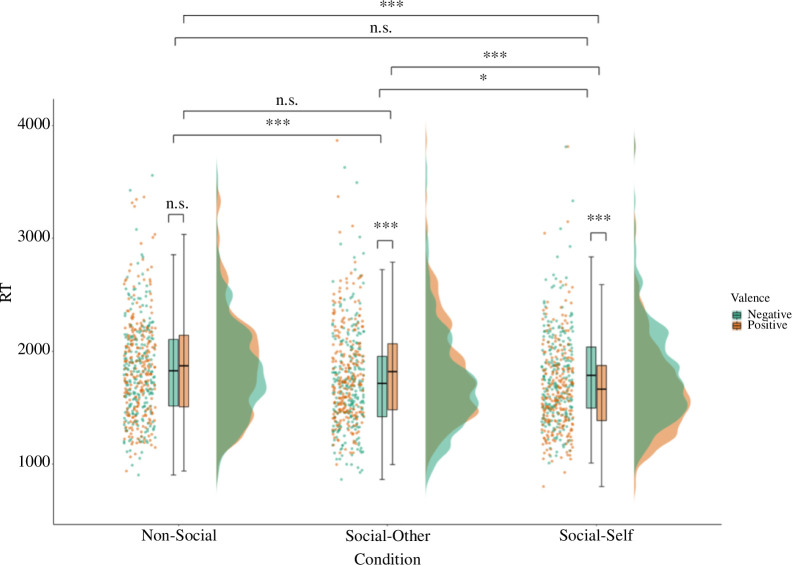
Network task performance depending on valence and condition. The figure illustrates the interacting effects of valence (negative versus positive) and condition (non-social versus social-other versus social-self) on reaction time. RT = Average reaction time on correct trials; Non-Social = trials measuring working memory for non-social relations (i.e. flight paths); Social-Other = trials measuring working memory for social relations in networks including only other individuals; Social-Self = trials measuring working memory for relations in social networks including the self and others; Negative = negative valence trials measured working memory for associations between individuals/airports that are not friends/have flights not running; whereas Positive = positive valence trials measured working memory for associations between individuals/airports that are friends/have flights running.

We next examined whether working memory for negative versus positive relations differed across social and non-social networks. In line with our prediction, there was a significant interaction between valence (negative versus positive) and condition (social versus non-social) for working memory reaction time (but not accuracy; table 2*d*). Looking at the effect of valence on working memory for non-social network relations showed a non-significant negativity bias (*β* = −0.10, 95% CI: −0.22 to 0.02, *t* = −2.15, *p* = 0.121) and no effect of valence in the social condition (*β* = 0.02, 95% CI: −0.06 to 0.10, *t* = 0.61, *p* = 0.956; electronic supplementary material, table S5A). However, further dissociating between social networks that included the self and those including only others (electronic supplementary material, table S5B), showed that the non-significant effect of valence in the social condition was due to opposing effects of valence in the social-self versus social-other conditions (table 2*e*; figure 3). Specifically, working memory was faster for negative compared with positive relations in the social-other condition (*β* = −0.24, 95% CI: −0.37 to −0.11, *t* = −5.25, *p* < 0.001), while the opposite effect was observed in the social-self condition where working memory was faster for positive compared with negative relations (*β* = 0.28, 95% CI: 0.15–0.41, *t* = 6.14, *p* < 0.001). When comparing the effect of valence across groups, the results showed that working memory recall for negative relations was faster in the social-other compared with the social-self (*β* = −0.16, 95% CI: −0.29 to −0.04, *t* = −3.57, *p* = 0.003) and non-social (*β* = 0.26, 95% CI: 0.13–0.38, *t* = 5.57, *p* < 0.001) conditions, with no significant difference between the non-social and social-self conditions (*p* = 0.343). This suggests that participants were fastest at accurately recalling other’s negative relations. For positive associations, working memory recall was faster in the social-self compared with the social-other (*β* = 0.36, 95% CI: 0.23–0.49, *t* = 7.83, *p* < 0.001) and non-social (*β* = 0.48, 95% CI: 0.35–0.60, *t* = 10.35, *p* < 0.001) conditions, with no significant difference between the non-social and social-other conditions (*p* = 0.098). This indicates that participants were fastest at accurately recalling self-relevant, positive relations.

### (d) Social working memory advantage across high and low cognitive load

In response to reviewers comments, we extended our pre-registered analyses to investigate whether the observed social working memory advantage would be uniform across low and high cognitive load (electronic supplementary material, table S6). For accuracy, load showed no significant interactions with condition or valence (electronic supplementary material, table S6). For reaction time, however, the effects of condition and valence varied as a function of cognitive load. Specifically, adding load to the model testing H1a, showed a significant interaction between condition and load for reaction time (*F* = 14.15, d.f. = 1200.00, *p* < 0.001). Unpacking this association showed that the effect of condition was significant under low, but not high, cognitive load (electronic supplementary material, table S7), with social-other and social-self network associations being recalled faster than the non-social condition (electronic supplementary material, table S8 and figure S3).

Adding load to the model testing H1b revealed a significant interaction between valence and load for RT (*F* = 64.65, d.f. = 2644.00, *p* < 0.001). The effect of valence was significant under both low and high cognitive load. However, the effect of valence was in opposite directions, such that a negativity bias was observed in low-load trials, while a positivity bias was observed in high-load trials (electronic supplementary material, table S9 and figure S4).

The model testing H1c, showed that the effects of condition and valence interacted with load. The significant three-way interaction (*F* = 7.63, d.f. = 2636.00, *p* < 0.001) was deconstructed to investigate the effect of valence in each condition for low and high load separately (electronic supplementary material, table S10). These post hoc comparisons showed that, while no significant interaction was observed between condition and valence on high load trials, for low load trials negative associations were recalled faster in the non-social and social-other condition, while positive associations were recalled faster in the social-self condition (electronic supplementary material, table S11 and figure S5). That is, the overall effects observed for reaction were driven by differences that emerged under low, not high, cognitive load.

### (e) Social and non-social working memory across levels of depression

Contrary to our hypothesis, levels of depression (high versus low) were not associated with slower (*p* = 0.483) or less accurate (*p* = 0.844) working memory overall (electronic supplementary material, table S12). There was also no evidence to support the prediction that higher levels of depression are associated with better or faster working memory for negative compared with positive relational information for social versus non-social networks (electronic supplementary material, tables S13–S15). In response to reviewer comments, we ran exploratory follow-up analyses where depression was entered into the model as a continuous variable, all results were in line with the results for depression as a dichotomous score. As per the pre-registration, all depression analyses were also run for negative and positive affect. The analyses yielded the same pattern of results as the depression effects and are therefore reported in the electronic supplementary material, tables S16–S23.

## 3. Discussion

Successfully navigating our social networks is important for maintaining enduring positive social connections, that provide benefits for human health and wellbeing. The current study hypothesized and provides evidence for a selective advantage of human working memory for recalling social versus non-social network information.

We developed and validated a novel NMT, capable of dissociating working memory performance for self-relevant and self-irrelevant social network information and information from non-social networks. Participants were tasked with accurately and quickly reporting on the relationships observed in each of these three network types. Our study extends previous work examining differences in learning about social and non-social networks (e.g. [11]) by probing differences in working memory for self-relevant and self-irrelevant social networks with non-social networks. Our study also extends the literature on social working memory (e.g. [8]), in two ways: first by explicitly examining working memory for relational social information (within networks) and second by examining this in direct contrast to non-social relational information.

Individuals were more accurate and faster at recalling information about social versus non-social networks. The superior recall of social over non-social information is in line with the social brain hypothesis [9] that our brain evolved to manage our increasingly complex social environments. This finding is also consistent with other work showing that social knowledge facilitates chunking in working memory. For example, in one study participants were more accurate at identifying changes in arrays of individuals facing (versus not facing) each other, if they depicted meaningful interactions [30]. This effect is also observed in young infants, who are better able to remember dolls when they are observed interacting (versus not interacting), which suggests that meaningful social interactions enhance memory through social chunking [31]. Therefore, in our study, it is possible that this mechanism enhanced participants’ ability to recall relationships within the social versus non-social networks. That said, our design did not show participants interacting *per se*, but rather the vignettes describing a social scenario in which actors interacted may have facilitated this effect.

It should be noted that the results observed in the present study could also be driven by greater expertise, rather than an evolutionary advantage, for social over non-social information. While individuals observe and are faced with multiple social interactions daily, their exposure to flight paths and connections may be more limited. This could in turn result in better memory for the more familiar (i.e. social) types of interactions. Future research should therefore investigate whether the same social memory advantage found in the present study can be observed in individuals who are equally familiar with interactions from the social and non-social conditions of this study (e.g. pilots). A further limitation of the paradigm is that it cannot determine at what point in working memory processing (i.e. encoding, maintenance, retrieval) the observed advantage for social compared with non-social networks arises. The task only measures performance at retrieval. Neuroimaging work using a social working memory task, shows unique patterns of activation for social working memory at both encoding and retrieval [32], suggesting that the social working memory advantage may be observed across all stages of working memory processing.

In our study, working memory for social networks further varied as a function of self-reference. The self-reference effect in episodic memory, refers to the phenomenon that people more reliably recall information that is in some way related to the self, compared with non-self-relevant information [33,34]. Beyond episodic memory, preliminary evidence shows a working memory advantage for self-relevant information [35–37], with a positivity bias in healthy individuals [38]. In line with these findings, participants in the current study recalled self-relevant social network information more accurately and faster relative to self-irrelevant social and non-social network information. Tracking ties within one’s own social networks is likely to be particularly relevant, as individuals’ understanding of social relations can inform their inferences about others’ behaviours and intentions as well as recognizing threats to existing social ties [39].

From an evolutionary perspective, this enhanced ability to recall self-relevant social information probably confers fitness advantages. This suggests that memory systems may have evolved to preferentially process, store and retrieve social information, particularly when it pertains to the self [40]. For example, studies have demonstrated that individuals who are more aware of their social status within a network are better able to manage their relationships and exert social influence [41,42]. Indeed, this advantage to recalling self-relevant information may reflect the need for individuals to assess the degree of influence they have over their social ties and the extent to which they are likely to be influenced by others in their network. This influence might play out across a number of different processes including the transfer of information and knowledge [43,44]. These findings are also especially relevant in the context of contemporary social interactions, which often occur in online social networks, where one’s position in a network is often explicitly quantified (e.g. through likes or comments on social media posts, or the ratio of followers to those an individual follows [45]).

Individuals were also faster at recalling positive self-relevant, versus negative self-relevant social network information, whereas social relations from networks including only others were remembered faster if they were negative. In other words, participants demonstrated a self-relevant positivity bias, but an ‘other’ negativity bias. Previous studies have also found a positivity bias, such that individuals more accurately recall positive, rather than negative, self-relevant information [46,47]. The current results are especially noteworthy given prior meta-analytic findings showing that among psychologically healthy individuals, emotional working memory effects are weak on behavioural measures of working memory [7], and more reliably observed at the neural level. These findings suggest that valence within a social context may more reliably influence working memory, relative to a non-social context.

The opposing finding showing faster retrieval of negative social information from social networks comprising only others is consistent with other work demonstrating the rewarding effects of observing the misfortune of others [48]. Alternatively, or additionally, knowing who is liked and disliked among other social networks can inform the success of one’s interactions when integrating with a new network of individuals. This is of particular importance given the human need to belong [49], and to identify risks to one’s social safety [25,50]. One possibility is that we place greater weight on who others dislike, rather than like, as befriending a disliked individual may become a risk to one’s own chances of group belonging. Importantly, further experimental work is needed to unpack the mechanisms influencing this *other* negativity bias, and to isolate how these processes may be modulated depending on factors such as one’s pre-existing social status. It is also possible that we did not observe a self-relevant negativity bias within our experimental paradigm as the networks we presented were imaginary, and therefore void to any risk of participants’ true social standing in their respective social networks. Alternatively, our findings might reflect inbuilt heuristics that drive human behaviour, such as the confirmation bias [51]. For example, individuals may remember information that confirms their pre-existing positive beliefs about themselves. Indeed the findings are in line with the commonly observed optimism bias [52].

In response to reviewer comments, we further examined whether our findings would hold across different levels of cognitive load (i.e. high versus low). These exploratory analyses revealed that cognitive load did not interact with condition or valence for working memory accuracy. That is, individuals were more accurate at recalling connections from social versus non-social networks irrespective of how many connections they were tasked with remembering. However, for reaction time the effects of condition and valence varied as a function of cognitive load. Individuals were faster at recalling information from social networks relative to information from non-social networks under low cognitive load, but not under high cognitive load. More nuanced findings emerged when we examined the interaction between condition, valence and cognitive load, such that negative associations were recalled faster in the non-social and social-other condition, while positive associations were recalled faster in the social-self condition, only under low load. Collectively, these findings suggest that our reaction time results, appear under low, but not high, cognitive load, whilst our accuracy results are consistent at different levels of cognitive load.

These results are in line with work showing that increasing cognitive load demands slows reaction time across a number of different tasks (e.g. in guided search [53] and driving-related tasks [54]). These findings are likely due to cognitive resources becoming increasingly taxed as cognitive load increases, subsequently slowing the speed with people can respond to stimuli. Interestingly, in one such study, completing a driving task with higher cognitive load demands improved accuracy during real world driving but slowed participants reaction time [54]. These findings echo ours, suggesting that alterations in cognitive load may differentially impact accuracy and reaction time performance, whereby reaction time is more sensitive to changes in cognitive load demands. It is possible that accuracy is less impacted as individuals enact compensatory mechanisms, such as increased effort, especially in situations where accuracy is valued. However, given these unplanned analyses were exploratory, it is important that future work seeks to replicate these observed effects.

Counter to our hypothesis, we found no effect of depression on task performance. This is in contrast to previous work showing executive functioning difficulties among individuals with depression [55,56]. Though it should be noted that we simply measured symptoms of depression, we did not compare individuals with clinical depression to healthy individuals. This may account for the lack of an effect of depression observed in the current study. Interestingly, depressive symptoms were not associated with a greater impact of social compared with non-social information on working memory processing. This is, in line with work showing a maintenance of an affective working memory advantage in depressed compared with non-depressed individuals [57]. It is in contrast, however, with the hypothesized heightened sensitivity to social information in individuals with elevated levels of depressive symptoms [25]. This may in part be accounted for by the fact that the task is not encoding individuals’ actual social relations and social status, thereby limiting the socio-affective salience of the information encoded in working memory [27]. Tracking working memory updating in real-world contexts instead may evidence greater individual differences in individuals with or at risk for depressive symptoms compared with psychologically healthy individuals.

In conclusion, our study presents evidence that human working memory selectively encodes social network information, and this is especially true when this information is self-relevant. We also reveal a more nuanced finding, that working memory for self-relevant social network information is biased towards positive information, while working memory about others’ social network relations is biased towards negative information. We argue that these effects facilitate the successful navigation of the complex social networks within which we all live. Networks that are critical for maintaining human health and wellbeing. Future work could extend our findings by comparing working memory for social and non-social relational information to non-relational information (i.e. memory for item characteristics that are beyond their relation to other items). We would hypothesize that relational information would be preferentially remembered over non-relational information given the causal mapping between constructs. Our findings also suggest that this effect would be potentiated for social information.

## 4. Methods

This study was pre-registered prior to participant recruitment (https://osf.io/4fcxn) and was approved by the University of New South Wales Human Research Executive Committee (HC Number 220233). All data and code are available here [58].

### (a) Participants

As per the pre-registration, participants were recruited until 244 individuals completed all parts of the study faithfully (did not fail more than 2 out of 5 attention check items included throughout the experiment). A sample size of 244 was calculated at a power of 0.80 and a significance threshold of *α* = 0.05, assuming a small-medium effect size (*f* = 0.2 [59]) to detect an interaction between condition (social-self, social-other and non-social), valence (positive versus negative) and depression (high versus low depressive symptoms). As one of the recruited participants failed three attention check items, this participant was excluded and another was recruited in their place, for a total recruitment of 245 participants.

To participate in the study, individuals had to be 18–65 years of age, be fluent in English, and have no history of head injury, neurological or neurodevelopmental disorders, or diagnosed learning disability. Additionally, to aid in the recruitment of participants with high versus low levels of depression, two different groups of participants were recruited. Half of the participants were recruited if they had reported no history of mental health illness, while the other half were recruited if they had reported a mental health illness. Screening was conducted at the recruitment stage using Prolific’s existing pre-screening filters to ensure participants met the inclusion and exclusion criteria prior to commencing the study. All participants who met these criteria provided informed consent prior to commencing the study and were awarded £8.75 through Prolific upon study completion (as per the platform’s guidelines on fair and equitable compensation).

Three participants were additionally excluded for performing below chance at the network task (responding accurately on less than 50% of the trials). The final sample comprised 241 participants. See table 1 for complete sample demographic characteristics.

### (b) Procedure

Following informed consent participants completed a set of questionnaires, an affective digit span task and the NMT. Participants were then compensated for their participation. The total completion time for the study was less than 1 h.

#### (i) Questionnaires

Participants answered standard demographics questions including age, gender, ethnicity and socio-economic status (SES).

*Depression.* Participants then completed the short Anxiety Depression Stress Scales (DASS-21) [60]. The DASS-21 assesses severity of symptoms of depression, anxiety and stress over the last week on a four-point Likert scale. Each item is rated from 0 (*never*) to 3 (*almost always*) with higher scores representing higher symptoms of depression, anxiety and stress. The scale demonstrated good internal consistency (*ω*_T_ = 0.95). Scores above 13 on the depression subscale of the DASS-21 were used as a cutoff to classify individuals with high versus low depressive symptoms, as this cutoff differentiates between individuals with normal/mild levels of depressive symptomatology and those with moderate and above levels.

*Negative and positive mood.* Participants’ mood was assessed with the Positive and Negative Affect Schedule (PANAS) [61]. The PANAS is a 20-item scale where participants indicate to what extent they experienced 10 positive/negative adjectives over the past two weeks. Each item is rated on a 5-point Likert scale ranging from 1 (*very slightly or not at all*) to 5 (*extremely*). Higher scores indicate greater positive/negative affect. The scale demonstrated good internal consistency (*ω*_T_ = 0.95).

#### (ii) Affective backward digit span task

The affective backward digit span task [62] was included to investigate its association with performance on the novel NMT. In this span task, participants were shown a series of digits superimposed over neutral (neutral condition) or mildly negative (affective condition) background images. Participants were required to ignore the background images and repeat the sequentially presented digits in reverse order.

#### (iii) Network memory task

In the novel NMT (figure 1), participants were presented with three conditions: social-self (social networks involving fictional relationships between the self (you) and 3–4 other individuals), social-other (social networks involving fictional relationships between Sam and 3–4 other individuals) and non-social (networks involving fictional flight paths between the city Hay and 3–4 other cities). Within each condition, participants were shown 12 different imaginary networks, 6 consisting of 4 individuals or cities (high cognitive load) and 6 consisting of 3 individuals or cities (low cognitive load). For each network, participants were first shown a vignette describing the relationships within the network for 7.5 s, before a network diagram of these relationships was also displayed for an additional 30 s (participants had the option to proceed after 15 s from diagram appearance). In these diagrams, individuals and cities were represented as nodes, with the relationships between them illustrated as edges. In each condition the names ‘You’ (social-self), ‘Sam’ (social-other) and ‘Hay’ (non-social) are included in each network, appearing in the exact same location. The three words are also matched in length in order to keep them as perceptually similar as possible.

Participants were then shown three individual connections from the network they had just seen and were asked to identify whether the two people shown were friends (social conditions) or whether flights were running between the two cities shown (non-social condition). In total, 36 connections were shown per condition. Half of the connections shown were positive (people were friends or flights were running), while the other half were negative (people were not friends or flights were not running). Immediately prior to commencing the main trials of the NMT (with order of condition presentation counterbalanced across participants), participants completed two practice rounds (one social and one non-social) following this same procedure.

Participant accuracy and reaction time for each trial of the network task were recorded. As per the pre-registration, accuracy performance on the network task was checked after the first 20 participants completed the experiment, as the NMT was a novel paradigm. Accuracy for these first 20 participants was above 65% and the study resumed as planned. Accuracy on the network task was identified as the correct classifications of a network association as positive versus negative in each trial. Total accuracy was computed by adding the number of correct classifications in each condition. Reaction time was operationalized as the average time taken to accurately classify the associations in each condition.

### (c) Analyses

All statistical analyses were conducted using R v. 4.0.1 (see electronic supplementary material for packages used). Unless otherwise specified, all reported statistics were based on linear mixed models including participant ID as a random effect. All models also included age as a pre-registered covariate, due to age-related differences in working memory performance [28] and social information processing [29]. To answer hypotheses 1a, 1c and 2c comparing social versus non-social conditions, social condition was indexed as average performance across the social-self and social-other conditions.

Hypothesis 1a was tested with condition (social versus non-social) entered as a predictor of reaction time and accuracy. Hypothesis 1b was tested with valence (positive versus negative) entered as a predictor of reaction time and accuracy, while hypothesis 1c was tested by including both condition (social versus non-social) and valence as predictors of reaction time and accuracy. Hypotheses 1a and 1c were additionally run with condition as a three-level factor (non-social versus social-other versus social-self), with contrast analyses run to investigate the relationships between the different conditions.

Hypothesis 2a was tested with depression (high versus low) entered as a predictor of reaction time and accuracy. To test hypothesis 2b, valence (positive versus negative) was added as a second predictor to the model described for 2a, and to test hypothesis 2c condition (social versus non-social) was additionally added as a third predictor to the model described for 2b. To test hypothesis 2d, condition was instead added as a three-level factor (non-social versus social-other versus social-self) to the model described for 2c.

As per the pre-registration, all predictions made under H2 for depression were also tested for negative affect and predicted reverse associations for positive affect (see electronic supplementary material for analyses output). Correlation analyses were run to check performance on the network task compared with performance on the affective backward digit span task. To help validate the network task, we expected to find a positive correlation between working memory effects in this task and in the affective backward digit span task.

To explore the effect of cognitive load on network task performance, analyses were also run with load (high versus low) entered as a predictor of reaction time and accuracy. We predicted that participants would classify the associations from the three (low load) versus four (high load) node networks more quickly and accurately, as cognitive load (the amount of information held in working memory) is substantially higher among the four-node networks, relative to the three. In response to reviewer comments, we ran further analyses with load added as a predictor to all models. As these additional analyses were not pre-registered, a Bonferroni-corrected significance level of *p* < 0.007 was applied for the seven additional outcomes (H1a–c, H2a–d).

Effect sizes were calculated for all primary analyses. As recommended for mixed effects model analyses, *r*^2^ values are reported [63].

## Data Availability

Our data and code can be found in the Dryad Digital Repository [58]. Supplementary material is available online [64].
